# Brain Tumour Detection Using VGG-Based Feature Extraction With Modified DarkNet-53 Model

**DOI:** 10.1155/ijbi/5535505

**Published:** 2025-05-30

**Authors:** S. Trisheela, Roshan Fernandes, Anisha P. Rodrigues, S. Supreeth, B. J. Ambika, Piyush Kumar Pareek, Rakesh Kumar Godi, G. Shruthi

**Affiliations:** ^1^Department of Computer Science and Engineering, Nitte Meenakshi Institute of Technology, Bengaluru, India; ^2^Department of Cyber Security, NMAM Institute of Technology, NITTE (Deemed to be University), Nitte, India; ^3^Department of Computer Science and Engineering, NMAM Institute of Technology, NITTE (Deemed to be University), Nitte, India; ^4^School of Computer Science and Engineering, REVA University, Bengaluru, Karnataka, India; ^5^Department of Computer Science and Engineering, Manipal Institute of Technology Bengaluru, Manipal Academy of Higher Education, Manipal, India; ^6^Department of AI&ML, Nitte Meenakshi Institute of Technology, Bengaluru, India; ^7^Department of Computer Science, Central University of Karnataka, Kalaburagi, India

**Keywords:** brain tumour, deep learning, invasive weed optimization, magnetic resonance imaging, modified DarkNet-53, VGGNet

## Abstract

The objective of AI research and development is to create intelligent systems capable of performing tasks and reasoning like humans. Artificial intelligence extends beyond pattern recognition, planning, and problem-solving, particularly in the realm of machine learning, where deep learning frameworks play a pivotal role. This study focuses on enhancing brain tumour detection in MRI scans using deep learning techniques. Malignant brain tumours result from abnormal cell growth, leading to severe neurological complications and high mortality rates. Early diagnosis is essential for effective treatment, and our research aims to improve detection accuracy through advanced AI methodologies. We propose a modified DarkNet-53 architecture, optimized with invasive weed optimization (IWO), to extract critical features from preprocessed MRI images. The model's presentation is assessed using accuracy, recall, loss, and AUC, achieving a 95% success rate on a dataset of 3264 MRI scans. The results demonstrate that our approach surpasses existing methods in accurately identifying a wide range of brain tumours at an early stage, contributing to improved diagnostic precision and patient outcomes.

## 1. Introduction

The brain is the nerve system's nerve centre and a crucial organ responsible for regulating all other body activities. One of the most fatal brain disorders is a brain tumour, which is produced by an irregular expansion of cell proliferation inside the brain and can damage any part of the brain. If left untreated for a lengthy period of time, the damage and dysfunctions caused by these tumours to the brain can be fatal. Every year, brain tumours are expected to rise by 5%, according to the WHO [1]. According to new research, brain tumours are the 10th largest cause of death worldwide. At least 18,600 people will lose their lives this year to a malignant brain or central nervous system (CNS) tumour, according to recent estimates [2]. Therefore, a patient's prognosis can be improved with prompt and precise tumour diagnosis.

Because the stage and kind of tumour affect the course of therapy, an opportune and precise diagnosis of a brain tumour is crucial to the patient's health. Tumours can be difficult to spot because of their wide variety of sizes, locations, and shapes [3]. The prognosis of a patient with a brain tumour may be negatively affected by a missed or delayed diagnosis. To identify brain tumours, doctors have traditionally relied on a visual analysis of medical imaging and exact tumour location tracking [4]. These diagnostic pictures were derived from PET, CT, and MRI (magnetic resonance imaging). Because MRI scans provide such high-quality pictures of soft tissues, radiologists and medical specialists commonly use them to diagnose brain tumours [5]. Manually diagnosing tumours using ocular inspection is sometimes difficult since the tumour borders are unclear due to the presence of healthy tissues in close proximity. This makes the process of manually detecting tumours laborious and prone to misdiagnosis. Tumour misidentification can also occur due to noisy images [6], which can be caused by factors such as acquisition procedures or differences in imaging equipment. Biopsies are often performed to determine if the tissue is cancerous or benign. Cancer diagnosis is a difficult and time-consuming process. Therefore, due to the complexity of present methodologies, automated technologies are replacing traditional methods [7, 8].

It is critical that brain tumours be identified and classified quickly so that patients can begin receiving therapy as soon as possible. Advances in technology have made it possible for doctors to use robots to provide better treatment for their patients [9]. As artificial intelligence advance, more studies are being presented to address the difficulties of medical picture identification [10]. These automated technologies help medical professionals accurately detect brain tumours and make informed decisions about treatment [11]. Since the human eye cannot tell the difference between the many shades of grey in MRI pictures, these systems are invaluable for picking up on even the most subtle of hue shifts. Managing and analysing massive volumes of medical data are challenges in the field of medical image investigation. However, the current infrastructure is unable to handle the dramatic expansion of medical data. Big data methods are being used by modern machine learning algorithms for the analysis of medical picture data [12]. The advent of cutting-edge technology, particularly machine learning and artificial intelligence, has had a profound effect on the healthcare sector by giving hospitals and clinics a reliable resource for obtaining second views. Wherever radiologists are attempting to reduce the likelihood of a biopsy or check tumour depth or kind [13], the most robust automated frameworks shine.

The Python programming language was used to device the application. Python's standard libraries are all available for use without a licence fee since they are based on free and open-source code principles. The presence of deep learning packages for the diagnosis of brain tumours further makes Python a desirable choice [14]. DL, a type of machine learning, is the approach of choice for image processing in this study; it employs artificial neural networks and data. Many research on the subject of brain tumour diagnosis and categorization can be found in the scientific literature. Wired and wireless connections give users access to data centres that collect information from many sources (e.g., the cloud and big data). In order to create a model description or machine learning scheme, however, traditional machine learning techniques require the feature vector to be retrieved first. This feature vector extraction requires domain experts. These activities are time-consuming and keep the specialist very occupied [15].

This is why these methods require human intervention or preparation before they can handle raw data. Great strides have been made by solving this issue that machine learning experts have had to deal with for years. This is possible due to the fact that deep networks execute the learning procedure on raw data, as opposed to the usual machine learning and image processing approaches [16]. In this post, specifically, we will be talking about these things: Because of issues like high similarity between benign and malignant tumour lesions, which can lead to misclassification, and the presence of irrelevant and redundant information in the features extracted from the images, the available MRI images are insufficient for training a good deep model. To solve these glitches, we offer a novel, fully robotic DL-based method for identifying brain tumours in MRI scans.

In this study, we have modified the introduction to include specific references to nontumour conditions alongside the discussion of brain tumours. This adjustment clarifies the distinction between the two and highlights the importance of accurate diagnosis not only for tumours but also to rule out other neurological issues. We aim to ensure that readers understand the full scope of challenges in brain health diagnostics.

➢ Gliomas: These tumours arise from glial cells, which support and protect neurons in the brain. The exact cause of gliomas is not well understood, but genetic factors and exposure to ionizing radiation have been implicated.
➢ Meningiomas: Originating from cord, meningiomas are often benign. Risk factors include genetic mutations, previous radiation therapy, and hormonal influences, particularly in women.
➢ Pituitary tumours: These tumours develop in the pituitary gland, which regulates various hormonal functions in the body. They can be influenced by genetic factors, such as Multiple Endocrine Neoplasia Category 1 (MEN1), and other unknown environmental factors.
➢ Metastatic tumours: These tumours originate from cancer cells that have spread to the brain from melanoma. The spread is facilitated by the bloodstream or lymphatic system.

The rest of the paper is prearranged as follows: “Related Works” discusses the impact on brain tumour detection, “Method and Materials” covers materials and approaches, “Results and Discussion” presents the findings, and “Conclusions and Future Enhancement” outlines conclusions and future directions.

## 2. Related Works

In order to categorise the RMB reconstructions into six different groups, Hossain et al. [17] suggest employing a Self-ONN to create a lightweight classifier they term the microwave brain image network (MBINet). At first, RMB pictures were captured to establish an image collection for an experimental antenna (SMBI) system. There are a total of 1320 images: 300 in the “normal” category, 215 in the “malignant” category, 200 in the “double benign” and “double malignant” categories, and 190 in the “single benign” and “single malignant” categories, respectively. The images were then preprocessed with resizing and normalization methods. The dataset was then augmented to provide 13,200 training photos per fold for fivefold cross-validation. Accuracy, precision, recall, *F*1-score, and specificity for six-class classification using original RMB pictures were 96.97%, 96.93%, 96.85%, 96.83%, and 97.95%, respectively, after the MBINet model was trained. Better classification results (almost 98%) were achieved by the MBINet model when compared to four Self-ONN models. As a result, the MBINet model may be employed in the SMBI system's tumour classification utilizing RMB pictures with confidence.

Brain tumour classification is challenging. However, Mehnatkesh et al. [18] offer a new evolutionary algorithm design that could help our upgraded version of ant colony optimization (ACO); IACO, which utilizes a differential evolution technique, is also proposed. This amalgamation of concepts is a great middle ground between solution variety and local optima when designing ResNet topologies based on DL. It improves optimization performance. A framework attained a precision of 0.98694, according to the test findings, which demonstrates the efficacy of our IACO-ResNet algorithm in automatically classifying brain tumours.

As previously mentioned, Nanda et al. [19] laid up a model for classification. The saliency-*K*-mean-SSO-RBNN strategy is an innovative hybrid saliency-*K*-mean segmentation method that takes advantage of the social spider optimization (SSO) approach in networks (RBNN). A hybrid saliency map was created using a *K*-mean cluster–based technique to split the tumour zone. Following image segmentation using methods including cosine transform, inverse difference moment (IDM), skewness, multiresolution component analysis, and kurtosis, the saliency map is used to emphasize the target picture's most intriguing features. After SSO is used to optimize the cluster centre, RBNN is used to process the feature vector for effective classification. We demonstrate a basic classification model that utilizes the RBNN with a Gaussian kernel. A new hybrid model and the saliency-*K*-mean-SSO-RBNN feature. We compare our results to those of existing systems and examine their specificity, accuracy, sensitivity, *F*1-score, MCC, and datasets. Using three different datasets, saliency-*K*-mean-SSO-RBNN achieved classification accuracy ranging from 96% to 92% to 94%.

In order to distinguish tumour patients from healthy individuals, Alturki et al. [20] employ 13 characteristics collected from deep convolutional layers in a voting classifier. In order to train a model, deep convolutional features are taken from first- and second-order characteristics of brain tumours. The accuracy with which tumour patients are separated from those without tumours can be improved by employing deep convolutional features. The suggested voting classifier, when used in conjunction with complex characteristics, achieves 99.9% accuracy. There is evidence that the suggested method is more precise than state-of-the-art approaches.

A clustering model optimized using Harris Hawks' CNN-based brain tumour identification from MRI has been reported by Kurdi et al. [21]. The suggested strategy is tested using a collection of normal and pathological brain MRIs available in the Kaggle dataset. The gathered photos are processed using the median filter to remove extraneous and inconsistent features, making it easier to deal with the pixels. By picking the seed locations in accordance with the fuzzification process, we may anticipate the areas of the tumour that will be impacted. The number of nodes used to classify the areas according to their connections is determined by the fuzzy set. The uncertainty associated with region segmentation is kept to a minimum by the fuzzy-based calculation. The efficient area search employed here significantly reduces the complication of identifying edge details. Finally, the convolute network is used to extract unique characteristics for classification. The HHO method allows the completely convolute layer to recognise the output by fine-tuning the parameter. Harris Hawks, an optimization method inspired by nature, reduces the number of false positives and maximises the tumour detection rate.

By combining a PA, a relative self-attention block, and an IFC layer, Tang et al. [22] have built a spinal convolution attention network (SpCaNet) to make efficient use of the pathogenic characteristics of brain tumours. When it comes to detecting brain tumours, our approach is less ponderous and more effective, needing more than three times as few parameters as in the SOTA model. Additionally, we propose the GAM strategy for training the SpCaNet model to address the issue of the inadequate generalization ability of the conventional stochastic gradient descent (SGD) technique. In terms of classification accuracy, GAM outperforms SGD. Experimental findings show that the suggested technique does well in classifying brain tumours, with an accuracy of 99.28%.

The application of DL in brain has led to significant advancements. Basha et al. [23] employed a Mask R-CNN combined with VGG-16 for effective tumour segmentation. Similarly, G.T. and S.K.K. [24] utilized a pretrained VGG-16 CNN model to tumours from MRI images, demonstrating high accuracy. Bourennane et al. [25] introduced a cubic SVM method for deep feature extraction, enhancing classification performance. Barman et al. [26] compared the CNN and VGG-16 models, finding that the latter significantly improves detection accuracy. These studies highlight the value of VGG-16 and DL representations in medical imaging.

An effective framework for brain tumour categorization (BTC) using a HieDNN classifier was proposed by Shajin et al. [27]. The photos used here are sourced from the Brats dataset. Savitzky–Golay denoising is used as a preprocessing step to reduce noise in the input pictures. GLCM is used to strip images of their identifying characteristics, such as texture details. HieDNN classifier is given the extracted pictures, which aids in labelling the brain images. MATLAB is used to actualize the intended system. The proposed method outperforms prior approaches, such as the use of a CNN for BTC based on MRI images (CNN-BTC), the use of a microscopic brain tumour detector with classification using a 3D CNN (3DCNN-BTC), and the use of a BTC using a hybrid deep autoencoder, by a margin of 31.14%, 16.09%, and 11.48%, respectively.

The study [28] focuses on addressing the complexities associated with the diagnosis and surgical resection of brain tumours using traditional methods employed by radiologists and clinical experts. These methods are often labor-intensive, error-prone, and time-consuming, with positional accuracy limitations that can be significant for brain cell analysis. To overcome these challenges, the authors propose that an accurate tumour localization is critical for enhancing diagnosis, prognosis, and treatment planning in glioblastoma patients. The study “Radiogenomic classification for MGMT promoter methylation status using multi-omics fused feature space for least invasive diagnosis through mpMRI scans” presents a novel approach to predict the MGMT status, a significant genetic subtype of glioblastoma, by integrating radiomic and genomic data [29].

The problem typically includes the following components:

➢ Dataset: collecting or acquiring a dataset of brain images with labelled tumour regions. The dataset should cover various patient populations to ensure the model's effectiveness and generalization.
➢ Preprocessing: preprocessing the brain images to enhance image quality, remove noise, and normalize intensities. Common preprocessing procedures include resizing, filtering, and normalization.
➢ Feature extraction: identifying tumours and nontumour areas in brain imaging by collecting useful features. There is a wide spectrum of feature extraction methods available, from basic statistical assessments to cutting-edge approaches like deep extraction with pretrained models.
➢ Model development: designing and training a machine learning or DL model that can learn the patterns and characteristics of brain tumour feature extraction. Popular approaches or hybrid models combining multiple architectures.

## 3. Method and Materials

In this research, we aim to enhance tumour discovery in MRI scans by leveraging progressive DL procedures. The proposed approach utilizes a modified DarkNet-53 architecture for feature extraction, optimized with the invasive weed optimization (IWO) algorithm to critical features from preprocessed MRI images. The process begins with image preprocessing to remove noise and standardize the dataset, followed by feature extraction using the modified DarkNet-53 model, which has been enhanced to improve the discovery of malignant brain tumours. The inclusion of IWO in the feature extraction phase allows for a more efficient search for optimal parameters, leading to better classification presentation. We assess the model's effectiveness using numerous evaluation metrics, including area under the curve (AUC). Our approach is validated on a dataset of 3264 MR images, where it achieved a success rate of 95%. The proposed methodology proves a significant development in detecting brain tumours at early stages compared to traditional models.

**Figure 1:**
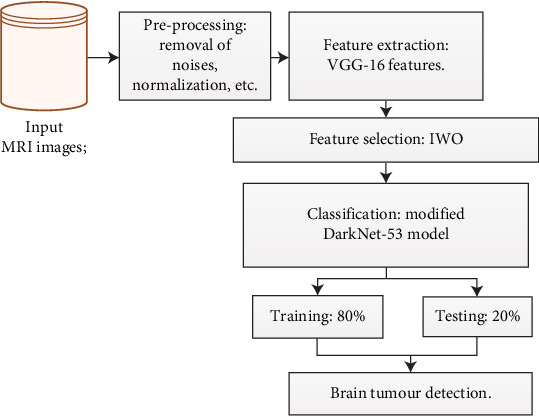
Overview of proposed flow diagram.

Figure 1 provides a high-level impression of the proposed methodology for brain tumour detection in MRI scans—the data acquisition and preprocessing, where MRI images are cleaned and standardized. Following this, feature extraction is performed using a modified DarkNet-53 architecture, optimized with the IWO algorithm—the classification stage, where the model predicts tumour presence. The diagram concludes with the AUC metric to assess the model's effectiveness.

### 3.1. Dataset Collection

**Figure 2:**
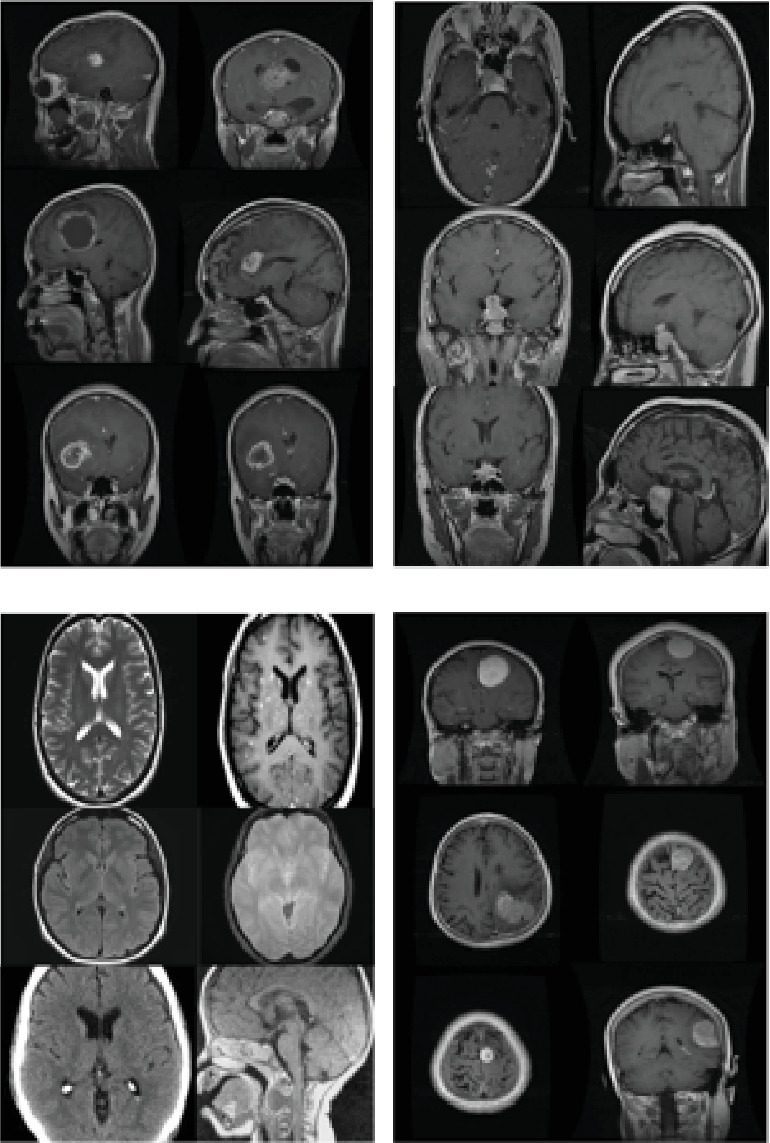
The brain tumours. MR images.

The dataset used in the study was composed from publically available sources on http://kaggle.com, with the goal of identifying brain tumours [30]. The dataset consists of images obtained through MRI scans. Because MRI is the most effective method for classifying brain cancers, we decided to use MR images in our investigation. We analyzed distinct types of brain tumours: meningioma (937 pictures) and no tumour (NT). Our dataset encompassed a total of 3264 MRI scans.  Figure 2 illustrates the MR images organized by brain tumour type, and Table 1 presents a breakdown of the dataset.

The dataset contains a higher sum of tumour images than normal (nontumour) images. This imbalance arises from the clinical emphasis on diagnosing and treating brain tumours, leading to more tumour cases being documented and available for study.

#### 3.1.1. Impact of Imbalance

An imbalanced dataset can lead to a bias towards the majority class (tumour cases), potentially resulting in higher false-positive rates for tumour detection and lower sensitivity to normal cases.

Imbalanced datasets can cause the model to learn an unequal distribution of features, making it more challenging to accurately classify underrepresented normal cases.

### 3.2. Preprocessing of the Dataset

In the preprocessing stage, the dataset is cleaned and filtered to ensure high-quality input for the classifier. This includes removing noise, normalizing image intensities, and resizing images to a consistent dimension suitable for our neural network. The MRI images are initially collected at various resolutions and sampling rates.

In order to make the data useful for training, preprocessing is a crucial step. Because they were pulled from a database of patients, the MR pictures were of poor quality and visibility. The term “preprocessing” refers to the steps taken after data collection to prepare it for analysis. It is a challenging process, and this part alone can often take more than half the total time needed to fix the issue. Most data collected are not in a useable condition at the time; thus, cleaning the data before using it to train the model is essential. The data must be preprocessed and filtered to remove information that is not relevant to categorization before it can be used.

In these cases, incomplete data is managed by adding the properties of interest required to carry out specific actions associated with fixing the problem. Traditional methods of data filtering, such as feature engineering, have their limitations [31]. The dataset is “cleaned” before being fed into any algorithm by removing any attributes and phrases that have nothing to do with categorization. At this point, any gaps in coverage are filled, redundant information is eliminated, and a usable data set is sent.

For our study, we standardized the sampling rate to 1 image per millimeter slice thickness, which is practiced in medical imaging to ensure consistency and adequate detail across the dataset. This rate was chosen to balance the need for detailed brain structure representation and manageable data size for efficient processing.

### 3.3. Data Division and Augmentation

By applying several transformations to the original data, data augmentation creates an artificially larger dataset. It is critical to think about and fix any possible side effects of augmentation, even though it can greatly improve the model's generalizability and resilience.

However, deep neural networks require a sizable dataset before they can deliver useful results, and ours only contained MR pictures. We employed a total of 3264 MR images in our dataset, allocating 80% to the training phase and the residual 10% each to testing and validation. The quantity of the original data impacts the training. The learning ability of the model is also improved by this. As a result, we used data augmentation by flipping the MR pictures horizontally and vertically and rotating, moving, and zooming the images. After that, we used the holdout validation method to ensure the accuracy of the datasets.

Benefits of data augmentation are as follows:

❖ Increased dataset size: By generating additional training samples, augmentation helps in reducing overfitting, particularly when the original dataset is small or imbalanced.
❖ Improved robustness: Augmentation techniques, scaling and zooming, introduce variability invariant features.
❖ Better generalization: Augmented data helps the classifier to generalize better to unseen data by simulating real-world variations and distortions.

### 3.4. Feature Extraction

Features are extracted from an image to capture crucial information for representation, such as shape, texture, and color. Optimal feature extraction from MRI is a formidable challenge. With the use of feature extraction, raw data may be transformed into interpretable numerical representations without losing any of the underlying information. Both DL and human-engineered feature extraction (HEFE) can be used to glean these characteristics. However, when done manually, feature extraction demands a thorough comprehension of which characteristics are most relevant to a given problem. It is sometimes easier to determine which traits could be helpful if one has a solid grasp of the context or topic. Extraction and selection procedures for best characteristics in photos, signals, audio/video, and text have been studied for years by scientists and researchers.

On the other hand, feature extraction is automated in DL frameworks without the need for human participation [32]. The proposed system uses the VGG-16 architecture's fully connected (FC7) layer to extract deep CNN features. Below, we list the specifics of the feature vectors that were retrieved.

#### 3.4.1. Deep VGG-16 Feature Extraction

DL is a learning paradigm that models complex data abstractions by simulating extensive networks of interconnected neurons through sophisticated architectures and nonlinear processes. Advances in data availability and computational power have driven researchers and businesses to explore increasingly intricate neural network topologies. DL has achieved notable achievement in various fields of artificial intelligence, particularly in speech and image classification.

One DL framework is the CNN [33], a type of feed-forward artificial human brain's neural structure. CNNs employ a combination of ANN principles and discrete convolutions to accurately extract features from data. They are particularly effective at processing and classifying 2D data, such as images and videos, by automating the feature extraction process directly from the input data. This capability is crucial for tasks like precise and reliable image recognition, including detecting brain tumours, where identifying subtle alterations in images is essential.

**Figure 3:**
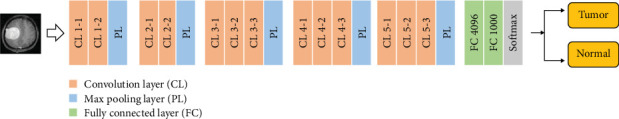
Architectural particulars of the VGG-16.

For this work, we selected VGGNet as the deep feature extractor. Introduced by Karen Simonyan and Andrew Zisserman in 2014 [34], VGGNet is renowned for its efficient feature extraction and lightweight design. It features 138 million limits and secured second place in the 2014 ILSVRC competition.  Figure 3 illustrates the internal structure of VGG-16, a variant of VGGNet. The framework processes 224 × 224-pixel images through a series of convolution layers (CLs), which use multiple filters to produce feature maps. Stacking several CLs enhances the model's ability to learn latent features, while activation functions, typically ReLU, are applied to maximize the output from these layers. The pooling layer (PL) follows, reducing the size of the feature maps to minimize processing power requirements for subsequent stages. The final fully connected (FC) layers generate class scores for classification. VGG-16 comprises 13 convolutional layers interspersed with 5 max-PLs, using 3 × 3 filters with a stride of 1. This contrasts with other CNN representations, such as ZF-Net and AlexNet, which use larger kernel sizes (7 × 7, 11 × 11) and higher strides in their early layers. VGG-16's choice of smaller kernel sizes helps preserve critical structural information in medical images while avoiding excessive parameter counts.

### 3.5. Feature Selection Using IWO

IWO is enthused by the weeds, which makes it effective for global optimization problems. It is particularly advantageous for feature selection due to its ability to explore and exploit the search space efficiently.

Global search capability: IWO can efficiently explore the global space, reducing in local minima.

Adaptability: The algorithm dynamically adjusts its limits, improving its adaptability to different datasets and feature spaces.

Convergence speed: IWO tends to converge quickly to an optimal solution, which is beneficial for computational efficiency.

Mehrabian and Lucas introduced (IWO) in 2006; it is a unique numerical stochastic optimization technique motivated by invasive weeds [35].

➢ Random initialization: Randomly initialize the populace *P* whose size is *P*_size_, where every separation is a vector with *d* dimension. In the reset procedure, there are some limits: the sum of the extreme iteration, iter_max_; the numbers of extreme seeds, *S*_max_; the statistics of least seeds, *S*_min_; nonlinear inflection index, *m*; the original value *σ*_initial_; and the final value *σ*_final_ optimization algorithms.
➢ Reproduction: The sum of seeds shaped by every weed is determined.
➢ Spatial dispersal: IWO uses usually dispersed random integers with zero mean and changing variance 2 to scatter the produced seeds over the search space. The deviation will be decreased from its starting value to its final value with each iteration. As described above, iter is the iteration:

(1) $$\begin{array}{c} \sigma_{\text{iter}}=\frac{{\left({\text{iter}}_{\max}-\text{iter}\right)}^{m}}{{\left({\text{iter}}_{\max}\right)}^{n}}\left(\sigma_{\text{initial}}-\sigma_{\text{final}}\right)+\sigma_{\text{final}}. \end{array}$$

By making this adjustment, the chances of a seed landing in a faraway location diminish in a nonlinear fashion at each time step, leading to a new population with higher fitness than the original. Because of this, the *r*-selection apparatus is now the *K*-selection instrument.

➢ Competitive exclusion: A plant will die out if it does not have any children, but it will spread everywhere. Therefore, the population is kept small to maintain plant-to-plant competition. After several repetitions of rapid germination and multiplication, the weed population has reached its maximum, pmax. Every weed undertakes reproduction and geographical dissemination according to the law of competitive survival. Next, the fitness values of the produced offspring and the weeds in the population are sorted from lowest to highest, and once the number of weeds is greater than *P*_size_, the weeds with the lowest fitness values are chosen as the novel population, and the remaining weeds are weeded out.

### 3.6. Classification Using Modified DarkNet-53 Classifier

The raw MRI data is first sent into the fine-tuned deep network VGGNet for training. The characteristics are extracted during training from the average global PL. In order to improve the extracted characteristics, an IWO model is used. At last, the modified features are fed into a final classification model, the modified DarkNet.

DarkNet-53 is a deep CNN with 53 layers. The YOLOv3 object detection architecture relies on this. It combines ResNet's strengths to guarantee supreme feature expression while staying away from the gradient issues the network might cause. It utilizes a hybrid of the residual network and the network. It is built from a series of convolution layers and residual blocks that are 1, 3, and 1 again. The following is a definition of the convolutional layer:

(2) $$\begin{array}{c} a_{m}^{n}=\underset{j\in X_{i}}{\sum }a_{j}^{n-1}*y_{jm}^{n}+z_{m}^{n} \end{array}$$

Equation (2) describes how multiple convolution kernels distort the input picture into *m* distinct feature maps, each of which is used to represent the image in layer *n*. The symbol ⁣^∗^. *x*_*j*_ represents the vector, while *y*_*jm*_ denotes *j*th kernel of mth layer. The second most important layer after the first layer is as follows:

(3) $$\begin{array}{c} a_{\text{out}}=\frac{\propto \left(a_{m}^{n}-\partial \right)}{\sqrt{\omega^{2}+\varphi }}+\gamma . \end{array}$$

The outcome of convolution computation is indicated by *a*_out_. The mean of the outputs is denoted by, variance of the input is denoted by, and is a constant offset signified by. *a*_out_ represents the end result of BN. Using batch normalization, the output is scaled so that its coefficients have the same distribution as the eigenvalues in the same batch. Afterwards, it features a convolutional layer that helps speed convergence and prevent overfitting. The subsequent layer incorporates a Leaky ReLu as an activation function in DarNet53. This operation makes network more nonlinear:

(4) $$\begin{array}{c} x_{j}=\left\{\begin{array}{cc} y_{j}, & \text{if }a_{\text{out}}\geq 0,\\ \frac{y_{j}}{b_{j}}, & \text{if }a_{\text{out}}<0. \end{array}\right. \end{array}$$

**Figure 4:**
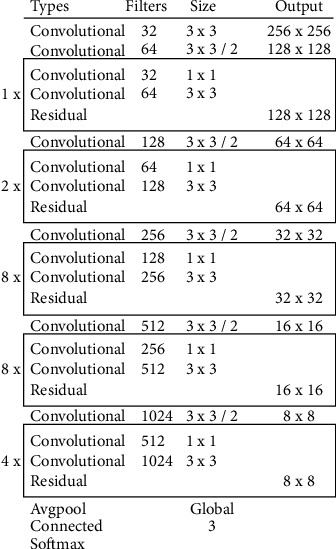
Modified DarkNet-53's deep model construction.

Equation (4) uses the variables *x*_*yij*_ and *B*_*j*_, which represent the input, activation, and fixed parameters, respectively, in the interval  [−1, +]. An additional critical component of this network is the PL. The network's weight sample size can be decreased using this layer. The final example demonstrates the possibility of combining features (weights) into a single layer. After that, the retrieved attributes are sorted into categories by the output layer. The sizes of input image are 256 × 256 pixels, and the model's parameters are 41.6 million. Its depth is 53, and its size is 155 MB.  Figure 4 displays the architectural details layer by layer.

PLs: Explanation of PLs: CNNs employ PLs to decrease the spatial maps that are inputted. The term for this procedure is “down-sampling,” and it helps to lower the network's computational load and parameter count. The pooling are average pooling and max pooling. The former takes the extreme value from each feature map region, and the latter takes the average. The model becomes more efficient and less susceptible to overfitting when PLs are used. These layers help to keep the most relevant features while eliminating the less significant ones.

#### 3.6.1. Transfer Learning (TL)

In our study, TL played an essential role in the finding of brain tumours from MRI scans. By employing a pretrained VGGNet model, we leverage its robust feature extraction capabilities, which have been honed on large-scale image datasets. This tactic allows us to significantly reduce training time and improve model accuracy while minimizing the risk of overfitting, particularly important given the limited size of medical imaging datasets. The hierarchical features captured by the VGGNet architecture enable our model to effectively perform detection.

##### 3.6.1.1. TL Role in Brain Tumour Detection:

TL, classically industrialised, is reused for one task as the preliminary point for a second, related task. In the context of brain tumour detection, transfer plays several key roles:

1. Leveraging pretrained models: By utilizing pretrained networks like VGGNet (trained on ImageNet), we can take advantage of the learned features. These models capture essential characteristics of images that are often useful in various applications, including medical imaging.
2. Feature extraction: TL allows us to extract hierarchical topographies from images effectively. The early layers of the network capture basic features patterns. This hierarchy is particularly valuable for distinguishing between normal and abnormal tissue in MRI scans.
3. Faster training: Since the model starts with weights that are already informed by previous training, it typically converges faster compared to scratch. This is especially beneficial when working with smaller datasets, common in medical imaging.
4. Reduced overfitting: With fewer training samples available, models are prone to overfitting. TL helps mitigate this by allowing the model to generalize better, as it benefits from the broad knowledge captured during the pretraining phase.
5. Improved performance: Ultimately, the application of TL enhances the representation's accuracy and robustness in detecting various types of brain tumours, making it a powerful tool in the diagnostic process.

TL is a method used in the classifier that has already been trained and is used to solve a new problem. Using previously learned tasks for freshly learned tasks can significantly enhance the supervised learning agent in practice. In formal mathematical terms, TL is defined as follows:

A domain *d* = {*Y*, *p*(*y*)} is labelled by two space *Y* and a delivery of marginal likelihoods *f*(*y*), where *y* = {*y*_1_, *y*_2_, *y*_3_, ⋯*y*_*n*_} ∈ *Y*. If there are two diverse areas, then they either have unlike marginal likelihoods (*p*(*Y*_*p*_) ≠ *p*(*Y*_*p*_)) or feature space (*Y*_*p*_ ≠ *Y*_*p*_).

Task: Given a specific domain *d*, mechanisms of task *t*{*X*, *g*(.)}, a space *X* and a prognostic function *g*(.), this is not noticeable, data {(*m*_*j*_, *n*_*j*_*j*{1, 2, 3, ⋯*N*}, where *m*_*j*_*Y* and *n*_*j*_*X*. From a probabilistic point, *f*(*m*_*j*_) as *p*(*n*_*j*_|*m*_*j*_); thus, we can revision the task *t* as *t* = {*X*, *P*(*x*|*Y*)}, if two tasks differ (*X*_*p*_ ≠ *X*_*q*_) or result in unlike distributions with provisional likelihoods (*p*(*X*_*p*_|*Y*_*p*_) ≠ *p*(*X*_*p*_|*Y*_*p*_)).

**Figure 5:**
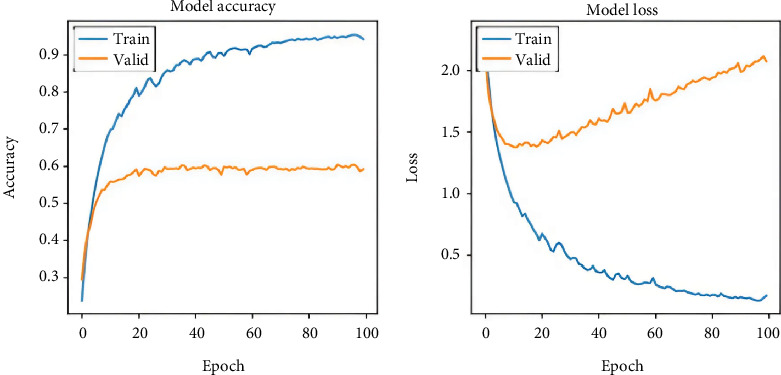
Validation accuracy and loss of the proposed model.

Transfer learning is depicted graphically in Figure 5. The deep model is changed to transfer the information of the original to the new domain. After that, we train this updated model using the subsequent hyperparameters: stochastic gradient algorithm with parameters of a learning degree of 0.001, 16, and entire 200 epochs. The GAP layer helps as basis for features. The retrieved characteristics are optimized using two reshaped *B*.

## 4. Results and Discussion

The classifier was developed in an environment called “Google Collaboratory” for the purpose of doing tests. A CNN-based model called “DarkNet” was developed with the help of this method. It is a well-known DL model used for accurate picture classification. Models were built using a variety of Python frameworks, including TensorFlow and Keras. “Binary Cross Entropy” was the loss function used by the auxiliary model.

We deployed our infrastructure using the entirely web-based Google Colab Pro+ platform. NVIDIA Tesla T4 GPUs, among others, were used in creation of the Google Colab Pro+ platform. This system also made use of a large amount of RAM (52 GB). Training machine infrastructure saves time and effort.

### 4.1. Validation Process

To validate the model effectively with the dataset comprising 3264 scan images, we employed a holdout validation method. This approach involves splitting the data into two subsets: 80% for training and 20% for testing. The holdout validation method is a widely used and reliable technique that divides dataset to evaluate the model's presentation. Specifically, the model was trained on the training subset, and its performance was then assessed on the testing subset. While the holdout method is effective, using only 20% of testing might affect the model's ability to oversimplify to new, unseen data. Consequently, the performance estimate could be biased if the testing subset does not fully represent the variability of the actual data.

### 4.2. Performances Analysis of Proposed Model With Existing Technique

In this study, we considered various groups of brain tumours, including gliomas, meningiomas, and pituitary tumours. Each type of tumour has distinct characteristics and potential causes:

➢ Gliomas: These tumours arise from glial cells, which support and protect neurons in the brain. The exact cause of gliomas is not well understood, but genetic factors and exposure to ionizing radiation have been implicated.
➢ Meningiomas: Originating from cord, meningiomas are often benign. Risk factors include genetic mutations, previous radiation therapy, and hormonal influences, particularly in women.
➢ Pituitary tumours: These tumours develop in the pituitary gland, which regulates various hormonal functions in the body. They can be influenced by genetic factors, such as MEN1, and other unknown environmental factors.
➢ Metastatic tumours: These growths begin as cancer cells that have metastasised to the brain. Metastasis to the brain occurs frequently in lung, breast, and melanoma malignancies, among others. The spread is facilitated by the bloodstream or lymphatic system.

Many metrics are utilised to assess the presentation of the proposed and existing models, including specificity, accuracy, sensitivity, *F*1-score, and the Matthews correlation coefficient.

True positive (TP) and false negative (FN) findings from binary classification are used to calculate these measures. Here is a rundown of performance measures and the corresponding mathematical formulas:

(5) $$\begin{array}{c} \text{Accuracy }\left(\text{AC}\right)=\frac{\text{sum of true postive and true negative}}{\text{sum of all values }}, \end{array}$$

(6) $$\begin{array}{c} \text{Specificity }\left(\text{Sp}\right)=\frac{\text{true negative }}{\text{sum of true negative and false positive }}, \end{array}$$

(7) $$\begin{array}{c} \text{Sensitivity }\left(\text{Se}\right)=\frac{\text{TP}}{\text{sum of TP and FN }}, \end{array}$$

(8) $$\begin{array}{c} \text{Matthew}{\text{s}}^{\prime }\text{ correlation coefficient }\left(M_{\text{CC}}\right)=\frac{1}{2}\left(1+\frac{\left[\text{TP}*\text{TN}\right]-\left[\text{FP}*\text{FN}\right]}{\sqrt{\left(\text{TN}+\text{FN}\right)\left(\text{TP}+\text{FN}\right)\left(\text{TN}+\text{FP}\right)\left(\text{TP}+\text{FP}\right)}}\right), \end{array}$$

(9) $$\begin{array}{c} F1‐\text{score}=\frac{\text{TP}}{\text{TP}+\left(1/2\right)\left(\text{FP}+\text{FN}\right)}. \end{array}$$

To compute the AUC values for all methods, we assume that the AUC of a model is typically estimated as in Equation (10):

(10) $$\begin{array}{c} \text{AUC}=\frac{\text{ sensitivity}+\text{specificity }}{2}. \end{array}$$

### 4.3. Validation Analysis of Proposed Model With Existing Techniques

The models' validation accuracy and loss are illustrated in Figure 5.

**Figure 6:**
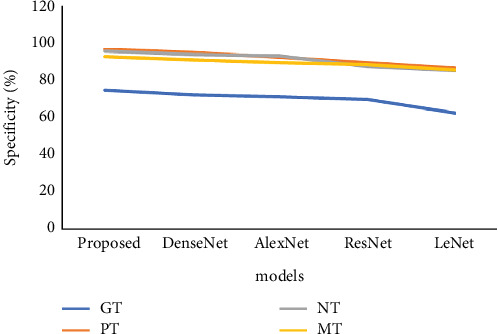
Analysis of specificity of proposed model vs. existing model.

Table 2 and Figure 6 represent the specificity analysis on various brain tumours. In this analysis, we used different analyses such as GT, PT, NT, and MT and also different methods. Initially, the modified DarkNet model reached the GT as 74.54, the PT as 96.44, the NT as 95.32, and finally the MT as 92.45, respectively. And another model of the DenseNet model reached the GT as 72.15, the PT as 94.39, the NT as 93.51, and finally the MT as 90.52, respectively. Next, the AlexNet model reached the GT as 70.93, the PT as 92.14, the NT as 92.92, and finally the MT as 89.13, respectively. Also, the ResNet model reached the GT as 69.56, the PT as 89.34, the NT as 87.12, and finally the MT as 88.30, respectively. And, finally, the LeNet model reached the GT as 62.45, the PT as 86.35, the NT as 85.09, and finally the MT as 85.24, respectively.

**Figure 7:**
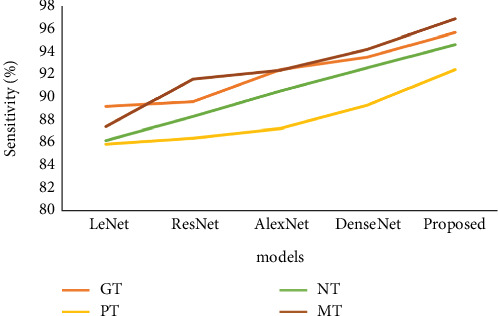
Analysis of sensitivity of the proposed model vs. existing model.

Table 3 and Figure 7 represent that the sensitivity analysis. In the sensitivity analysis, we have used different parameters and methods. Initially, the LeNet model reached the GT as 89.24, the PT as 85.92, the NT as 86.23, and, finally, the MT as 87.44, respectively. Also, the ResNet model reached the GT as 89.64, the PT as 86.45, the NT as 88.32, and, finally, the MT as 91.64, respectively. Also, the AlexNet model reached the GT as 92.45, the PT as 87.26, the NT as 90.58, and, finally, the MT as 92.43, respectively. Also, the DenseNet model reached the GT as 93.56, the PT as 89.35, the NT as 92.64, and, finally, the MT as 94.25, respectively. Also, the proposed model reached the GT as 95.71, the PT as 92.47, the NT as 94.65, and, finally, the MT as 96.89, respectively.

**Figure 8:**
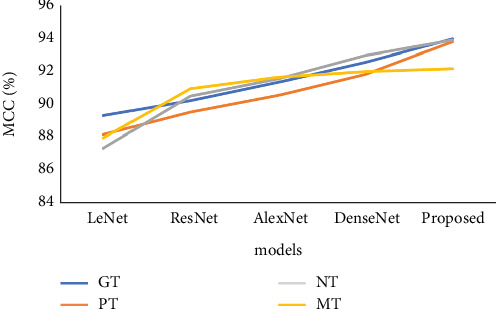
Analysis of MCC of the proposed classical vs. existing model.

We used various parameters and methodologies to represent the validation analysis of MCC in Table 4 and Figure 8. The LeNet model initially arrived at the GT as 89.34, the PT as 88.19, the NT as 87.34, and finally the MT as 87.96. The GT, PT, NT, and MT were all determined by the ResNet model to be, respectively, 90.23, 89.54, 90.53, and 90.98. Additionally, another method of the AlexNet model arrived at the GT, PT, NT, and MT as 91.34, 90.57, 91.56, and 91.67, respectively. The following DenseNet model reached the GT, PT, NT, and MT at 92.56, 91.83, 92.99, and 92.01, respectively. Additionally, according to the proposed model, the GT was 94.01, the PT was 93.82, the NT was 93.92, and the MT was 92.18. The modified DarkNet-53 deep replica features a layer-wise construction, similar to the DarkNet-53 deep model's layer-wise architecture.

**Figure 9:**
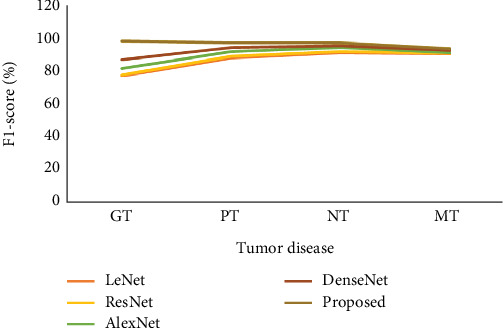
Analysis of *F*1-score of the proposed classical vs. existing model.

Table 5 and Figure 9 represent the *F*1-score analysis. In the *F*1-score analysis, we have used different parameters and methods. Initially, the LeNet model reached the GT as 76.56, the PT as 87.67, the NT as 90.66, and, finally, the MT as 90.00, respectively. After that, the ResNet model reached the GT as 77.33, the PT as 88.72, the NT as 91.34, and, finally, the MT as 90.24, respectively. Next, the AlexNet model reached the GT as 81.34, the PT as 91.54, the NT as 93.87, and, finally, the MT as 90.89, respectively. Also, the DenseNet model reached the GT as 86.45, the PT as 93.78, the NT as 95.02, and, finally, the MT as 92.13, respectively. And finally, the proposed model reached the GT as 97.88, the PT as 96.54, the NT as 96.62, and, finally, the MT as 93.12, respectively. In the analysis metrics, the proposed model reached better results than other compared representations.

**Figure 10:**
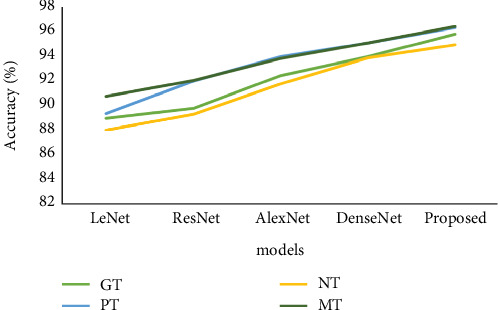
Analysis of accuracy of the proposed model vs. existing model.

Table 6 and Figure 10 display the accuracy analysis. For the *F*1-score analysis, we used a variety of parameters and techniques. The GT, PT, NT, and MT were all initially determined by the LeNet model to be 88.89, 89.23, 87.90, and 90.64, respectively. The GT, PT, and NT values that the ResNet model obtained were 89.67, 91.84, 89.18, and 91.89, respectively. The GT, PT, NT, and MT were all reached as 92.30, 93.79, 91.63, and 93.68 on the following AlexNet model, respectively. Following that, the DenseNet model arrived at the GT, PT, NT, and MT at 93.82, 94.92, 93.71, and 94.90, respectively. And finally, according to the proposed model, the GT was 95.64, the PT was 96.12, the NT was 94.78, and the MT was 96.24. The suggested model outperformed other compared models in the analysis metrics.

**Figure 11:**
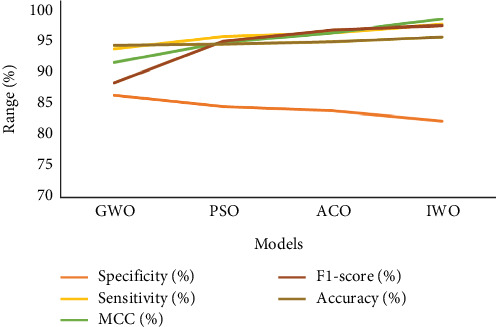
Analysis of feature selection of the proposed model vs. existing model.

Comparative analysis of different feature selection methods—GWO (grey wolf optimization), PSO (particle swarm optimization), ACO, and IWO—evaluated across six key performance indicators sensitivity, specificity, Matthews' correlation coefficient (MCC), *F*1-score, accuracy, and AUC, is depicted in Table 7 and shown using the graphical representation as in Figure 11.

❖ IWO stands out with the highest sensitivity (97.62%), *F*1-score (97.46%), accuracy (95.67%), and MCC (98.52%), indicating it excels in correctly identifying TPs while maintaining balanced classification decisions. However, it has the lowest specificity (82.21%) and slightly lower AUC (89.92%), suggesting a modest compromise in true negative detection and overall discriminatory power.
❖ ACO offers a near-optimal trade-off, scoring the highest *F*1-score (96.78%) next to IWO and slightly higher AUC (90.07%), though it performs lower in specificity (83.82%) and MCC compared to IWO.
❖ PSO demonstrates balanced performance, with second-best specificity (84.50%), strong sensitivity (95.75%), and competitive accuracy (94.47%) and AUC (90.13%), making it a solid middle-ground choice with relatively even strengths.
❖ GWO, while performing modestly across all metrics, has the highest specificity (86.25%), suggesting it is more conservative in identifying negative cases, but it lags behind others in sensitivity and *F*1-score.

Comparative evaluation between the proposed model and an existing technique referenced as Shoaib et al. [36], using five key performance indicators, specificity, sensitivity, accuracy, *F*1-score, and AUC, is depicted in Table 8. The existing method in all metrics except sensitivity, where Shoaib et al. [36] achieved a slightly higher value of 97.72% compared to 97.62% in the proposed approach—a marginal difference of 0.10%, suggesting both models are equally competent in identifying positive cases. However, the proposed model demonstrates substantial improvements in other areas, with specificity increasing to 82.21% (from 82.14%), indicating a better true-negative rate. More notably, the accuracy surges to 95.67%, significantly higher than the 93.15% achieved by Shoaib et al., reflecting improved overall classification performance. An *F*1-score of 97.46% for the proposed model far exceeds the 81.74% of the baseline, indicating much better result. Additionally, the AUC reaches 89.92%, highlighting the superior discriminative ability of the proposed method associated to the 81.74% in the existing technique.

### 4.4. Merits and Complexity of Proposed Model

#### 4.4.1. Merits

Our proposed method, which combines an enhanced DarkNet-53 architecture with VGG-based feature extraction and IWO, achieves a high accuracy rate of 95% on tested dataset. This superior performance indicates effective tumour detection and classification capabilities.

#### 4.4.2. Complexity

The integration of the enhanced DarkNet-53 architecture and VGGNet involves complex neural network structures with numerous layers and parameters. Training these models requires significant learning. This is depicted as below.

➢ VGG-16 feature extraction

For a given convolutional layer *l* in VGG-16

1. Number of filters: *F*_*l*_
2. Filter size: *K* × *K*
3. Input feature map size: *H*_*l*−1_ × *W*_*l*−1_ × *D*_*l*−1_
4. Output feature map size: *H*_*l*_ × *W*_*l*_ × *F*_*l*_

The computational complexity for one convolutional layer *l* is Equation (10):

(11) $$\begin{array}{c} O\left(H_{l-1}\times W_{l-1}\times D_{l-1}\times F_{l}\times K^{2}\right). \end{array}$$

➢ Modified DarkNet-53

DarkNet-53 contains 53 convolutional layers, with modifications potentially impacting the number of filters and layer connections.

For a given convolutional layer *m* in DarkNet-53

1. Number of Filters: *F*_*m*_
2. Filter Size: *K* × *K* (commonly 3 × 3 or 1 × 1)
3. Input Feature Map Size: *H*_*m*−1_ × *W*_*m*−1_ × *D*_*m*−1_
4. Output Feature Map Size: *H*_*m*_ × *W*_*m*_ × *F*_*m*_

The computational complexity for one convolutional layer *m* in DarkNet-53 is Equation (12):

(12) $$\begin{array}{c} O\left(H_{m-1}\times W_{m-1}\times D_{m-1}\times F_{m}\times K^{2}\right). \end{array}$$

➢ Combined model complexity

When combining VGG-16 for feature extraction and DarkNet-53 for further processing, the overall complexity can be represented as the sum of the complexities of the individual components.

If *L* represents the total sum of layers in VGG-16 and *M* characterises the total number of layers in modified DarkNet-53, then we have Equation (12):

(13) $$\begin{array}{c} O\left(\overset{L}{\underset{l-1}{\sum }}\;H_{l-1}\times W_{l-1}\times D_{l-1}\times F_{l}\times K^{2}\right)+O\left(\overset{M}{\underset{m-1}{\sum }}\;H_{m-1}\times W_{m-1}\times D_{m-1}\times F_{m}\times K^{2}\right) \end{array}$$

## 5. Conclusions and Future Enhancement

Brain tumours have a mortality rate, but early and accurate diagnosis can meaningfully progress patient outcomes. Due to variations in tumour shape, size, and structure, precise classification remains challenging. This study utilizes MRI scans for tumour segmentation, following a structured approach: preprocessing MRI data, extracting features with VGGNet, selecting optimal features using the IWO model, and classifying tumours with the DarkNet-53 DL model. Our method was compared with other machine learning models, demonstrating superior performance with 95.67% accuracy, 97.46% *F*1-score, 97.62% sensitivity, 82.21% specificity, and 89.92% AUC. A key limitation was the computational time required for training due to GPU constraints, which was mitigated by hardware optimization. Future work will focus on expanding the dataset, developing a specialized CNN model for brain tumour classification, and collaborating with medical professionals for real-world implementation. Moreover, data augmentation procedures such as rotation, scaling, translation, and flipping will be practical to improve model generalization. Fine-tuning and hyperparameter optimization, including Bayesian optimization, will further enhance performance, ultimately aiming for clinical adoption.

## Figures and Tables

**Table 1:** Dataset.

**Brain tumour**	**Type count**
Pituitary tumour (PT)	901
Glioma tumour (GT)	926
Meningioma tumour (MT)	937
No tumour (NT)	500
Total	3264

**Table 2:** Specificity analysis on various brain tumours.

**Method**	**GT**	**PT**	**NT**	**MT**
Modified DarkNet	74.54	96.44	95.32	92.45
DenseNet	72.15	94.39	93.51	90.52
AlexNet	70.93	92.14	92.92	89.13
ResNet	69.56	89.34	87.12	88.30
LeNet	62.45	86.35	85.09	85.24

**Table 3:** Sensitivity analysis.

**Method**	**GT**	**PT**	**NT**	**MT**
LeNet	89.24	85.92	86.23	87.44
ResNet	89.64	86.45	88.32	91.64
AlexNet	92.45	87.26	90.58	92.43
DenseNet	93.56	89.35	92.64	94.25
Proposed	95.71	92.47	94.65	96.89

**Table 4:** Validation analysis of MCC.

**Method**	**GT**	**PT**	**NT**	**MT**
LeNet	89.34	88.19	87.34	87.96
ResNet	90.23	89.54	90.53	90.98
AlexNet	91.34	90.57	91.56	91.67
DenseNet	92.56	91.83	92.99	92.01
Proposed	94.01	93.82	93.92	92.18

**Table 5:** *F*1-score analysis.

**Method**	**GT**	**PT**	**NT**	**MT**
LeNet	76.56	87.67	90.66	90.00
ResNet	77.33	88.72	91.34	90.24
AlexNet	81.34	91.54	93.87	90.89
DenseNet	86.45	93.78	95.02	92.13
Proposed	97.88	96.54	96.62	93.12

**Table 6:** Accuracy analysis.

**Method**	**GT**	**PT**	**NT**	**MT**
LeNet	88.89	89.23	87.90	90.64
ResNet	89.67	91.84	89.18	91.89
AlexNet	92.30	93.79	91.63	93.68
DenseNet	93.82	94.92	93.71	94.90
Proposed	95.64	96.12	94.78	96.24

**Table 7:** Feature selection analysis.

**Methods**	**Specificity (%)**	**Sensitivity (%)**	**MCC (%)**	**F**1** -score (%)**	**Accuracy (%)**	**AUC (%)**
GWO	86.25	93.75	91.50	88.25	94.32	90.00
PSO	84.50	95.75	94.75	95.00	94.47	90.13
ACO	83.82	96.31	96.34	96.78	94.89	90.07
IWO	82.21	97.62	98.52	97.46	95.67	89.92

**Table 8:** Comparison evaluation of the proposed model with existing techniques.

**Methods**	**Specificity (%)**	**Sensitivity (%)**	**Accuracy (%)**	**F**1** -score**	**AUC**
Shoaib et al. [36]	82.14%,	97.72%	93.15%	81.74%	81.74%,
Proposed model	82.21	97.62	95.67	97.46	89.92

## Data Availability

The labelled datasets used to support the findings of this study can be obtained from the corresponding author upon request.
